# Adverse Pregnancy Outcomes of Patients with History of First-Trimester Recurrent Spontaneous Abortion

**DOI:** 10.1155/2017/4359424

**Published:** 2017-07-17

**Authors:** Jing Yang, Yan Wang, Xiao-ye Wang, Yan-yu Zhao, Jing Wang, Yang-yu Zhao

**Affiliations:** ^1^Obstetrics & Gynecology Department, Peking University Third Hospital, Beijing 100191, China; ^2^Obstetrics & Gynecology Department, Peking University International Hospital, Beijing 102206, China

## Abstract

Although a history of first-trimester recurrent spontaneous abortion (FRSA) is regarded as a risk factor in antenatal care, the characteristic of subsequent pregnancy outcome is not clearly elucidated. Here, a retrospective analysis was performed on the clinical data of 492 singleton pregnant women. 164 of them with the history of FRSA were enrolled in study group, compared to 328 deliveries without the history of FRSA. For maternal outcomes, patients in the study group delivered earlier with mean gestational age and the incidences of cesarean section and postpartum hemorrhage were higher compared to the control group. For placental outcomes, the incidence of placenta-mediated pregnancy complications (PMPC) in the study group increased in terms of late-onset preeclampsia, oligohydramnios, early-onset fetal growth restriction, and second-trimester abortion. Patients in the study group were more likely to suffer from placenta accreta, placenta increta, and placenta percreta. For perinatal outcomes, the proportion of birth defects of newborns in the study group was greater. At last, logistic regression analyses showed that the history of FRSA was an independent risk factor for cesarean section and pregnancy complications. In conclusion, women with the history of FRSA are often exposed to an elevated incidence of maternal-placental-perinatal adverse pregnancy outcomes.

## 1. Introduction

Recurrent spontaneous abortion (RSA) refers to the consecutive occurrence of fetal loss (body weight < 1000 g) happening more than 2 times before 28 gestational weeks with the same sex partner [1]. The incidence ranges from 2% to 4% [2], of which nearly 80% appear in the first trimester. First-trimester RSA (FRSA) occurs within 12 weeks of pregnancy and its pathogenesis is more complicated than second-trimester RSA. No etiologic factor is identified in approximately 50% of FRSA cases. Previous studies have revealed that women with a history of RSA are exposed to higher rates of adverse maternal and fetal outcomes in their subsequent pregnancies [3–5], but few studies have focused on FRSA. Due to high incidence and complex etiology of FRSA, it is of great importance to investigate the subsequent pregnancy outcomes in order to ensure an effective perinatal care. In this study, we analyzed the associations between maternal-placental-perinatal pregnancy outcomes and history of FRSA. No intervention was administered during their pregnancies. The aim was to provide better therapies for future perinatal care.

## 2. Materials and Methods

### 2.1. General Information

A retrospective population-based analysis of all singleton pregnant women who gave birth at Peking University Third Hospital between January 2010 and September 2013 was performed. Total delivery number was 12811 during this period. All clinical records/information were anonymized and deidentified prior to analysis. Comparisons were made between women who have and have not undergone unexplained FRSA in order to estimate the relationship between FRSA and adverse pregnancy outcomes.

### 2.2. Inclusion Criteria

All patients in the study group met the following criteria: patients who had ≥2 abortions during early pregnancy. Furthermore, chromosome karyotypes of the couples were normal, and patients had no abnormal reproductive tract anatomy. Thyroid, endocrine functions and the insulin level were normal. In addition, TORCH IgM was negative, antiphospholipid and antinuclear antibodies were negative, and the coagulation function was normal. Furthermore, cervical secretion was negative. The couples were also found to be negative for hepatitis B, hepatitis C, syphilis, AIDS, or other infectious diseases. In addition, blocking antibodies was not detected among patients with normal results in the above examinations. A total of 164 cases were included. From 12647 cases without the history of FRSA and delivering in the same period, 328 patients were randomly selected at 1 : 2 ratio as the control group.

### 2.3. Exclusion Criteria

Exclusion criteria of all subjects included medical complications such as chronic hypertension, chronic kidney diseases, autoimmune diseases, and severe late-term pregnancy complications transferred from other hospitals.

### 2.4. Data Collection

Data were collected from the computerized perinatal database of the hospital, which comprised obstetric and perinatal information recorded directly after delivery by three obstetricians. Xiao-ye Wang and Yan-yu Zhao examined the information routinely before entering it into the database. Coding was done after assessing the medical prenatal care records as well as the routine hospital documents. These procedures assured the completeness and accuracy of the database. The following clinical characteristics were evaluated: gravidity, spontaneous abortion times, proportion of primiparous women, body mass index (BMI), gestational age at delivery, birth weight, the use of assisted reproductive therapy, and times of other adverse pregnancy histories. The following maternal complications were examined: the cesarean section and postpartum hemorrhage. Placental-derived disease including PMPC and placental diseases were assessed. PMPC refers to a group of maternal complications caused by insufficient placental implantation and placental ischemia, including preeclampsia, fetal growth restriction, placental abruption, spontaneous premature birth, and intrauterine fetal death [6–8]. Preeclampsia (PE) was subdivided into early-onset and late-onset PE according to the incidence of gestational age. 32 weeks was identified as the diagnostic cutoff between early-onset FGR and late-onset FGR [9, 10]. No intrauterine fetal death occurred in both groups. Preterm birth was defined as the birth of a live baby at <37 completed weeks of gestation. Very preterm birth was defined as the birth of a live baby at <32 weeks of gestation. Oligohydramnios caused by fetal malformations and maternal premature rupture of membranes was excluded. Placental-derived disease refers to placenta previa, placenta accreta, placenta increta, and placenta percreta. The following perinatal outcomes were assessed: small for gestational age (SGA), perinatal death, severe asphyxia neonatorum, and birth defects of newborns.

### 2.5. Statistical Analysis

Data were analyzed using Statistical Package for Social Sciences (SPSS version 19.0). Continuous data of normal distribution was presented as mean and standard deviation (mean ± SD). Independent sample *t*-test was used for comparison between the two groups. Chi-square test was performed to ascertain differences in qualitative variables. A multivariate logistic regression analysis was carried out in a backward elimination fashion to analyze the relationship between FRSA and adverse outcomes. *P* < 0.05 was considered to be statistically significant.

## 3. Results

### 3.1. Baseline Characteristics of the Study Population

The study population included a total of 12811 singleton patients who delivered at Peking University Third Hospital during nearly 3-year study period, of which 164 (1.28%) met the inclusion criteria. A total of 164 included cases remained for analysis. Gravidity and maternal age were significantly higher in the study group, while the gestational weeks at delivery and birth weight of newborns were less (Table 1).

### 3.2. Maternal Pregnancy Outcomes

Maternal pregnancy outcomes are presented in Table 2. There were significantly higher rates of cesarean section and postpartum hemorrhage.

### 3.3. Placenta Diseases

Placenta diseases are summarized in Table 3. PMPC such as the late-onset PE, oligohydramnios, early-onset FGR, and second-trimester abortion were significantly higher in the study group. There was no significant difference between the two groups in the incidence of early-onset PE, late-onset FGR, placental abruption, and early and late preterm delivery. The incidences of placenta diseases such as placenta accreta, placenta increta, and placenta percreta were significantly higher in the study group (*P* < 0.05); the difference of placenta previa rate between the two groups was of no significance. Considering that maternal age could cause the different incidence of late-onset PE, we then performed a multivariate logistic regression analysis in adjusted model and found that the incidence of late-onset PE was also significantly higher in the study group (Table 4).

### 3.4. Adverse Fetal and Neonatal Outcomes

Adverse perinatal outcomes are presented in Table 5. The proportion of birth defects of newborns in the study group was significantly higher as compared to the control group. There was no significant difference in the incidence of small for gestational age, perinatal death, and neonatal severe asphyxia between two the groups.

### 3.5. Adverse Pregnancy Outcomes Related to Recurrent Spontaneous Abortions

Using a multivariate regression analysis, with the following conditions as the outcome variable, respectively, controlling for corresponding confounding variables, FRSA was found as an independent risk factor for the following adverse pregnancy outcomes: cesarean section (OR = 1.7; 95% CI: 1.06–2.73; *P* < 0.05) and placenta diseases including PE (OR = 3.69; 95% CI: 1.87–7.30; *P* < 0.001), oligohydramnios (OR = 4.62; 95% CI: 1.84–11.64; *P* = 0.001), and second-trimester abortion (OR = 11.71; 95% CI: 3.36–40.81; *P* < 0.001) (Table 6).

## 4. Discussion

Most experts believe that women with a history of two or three consecutive spontaneous abortions have similar abortion risks in their subsequent pregnancies. Patients who experienced more than 2 incidents of miscarriage are often exposed to elevated incidences of placental dysfunction disorders and cesarean section [3, 11]. Such patients should be considered as high risk obstetric population. Their characteristics of pregnancy complications and the preventive measures should be investigated due to the necessity and critical clinical value. However, limitation of current studies cannot exclude confounding effects of other pathologies when examining pregnancy outcomes considering complex causes of recurrent miscarriage. The unexplained miscarriage group is the best group minimizing other confounding factors and therefore it is the most appropriate group [5]. In this study, only patients who experienced two or more unexplained spontaneous abortions were enrolled; they also received no special perinatal interventions during obstetric examinations, which can minimize the impact of different abortion causes and perinatal treatments. The characteristics of adverse pregnancy outcomes were studied from a placental-perinatal-maternal perspective. We found that placental-derived pregnancy-related complications and cesarean section rate were significantly correlated with FRSA history. Specific perinatal care and corresponding preventions of placenta diseases can be implemented to reduce the incidence of adverse pregnancy outcomes.

### 4.1. Placenta-Derived Diseases

In previous studies, RSA has been reported to have a similar pathogenesis to that of placental dysfunction complications, such as preeclampsia, intrauterine fetal death, oligohydramnios, small for gestational age children, placental abruption, and spontaneous preterm birth. It also correlates with placental ischemia caused by placenta implantation insufficiency during the first trimester [12–14], immune imbalance of Thl/Th2 and Thl7/Treg on maternal-fetal interface, insufficient invasion of extravillous trophoblastic cells, and uterine spiral artery dysfunction. Although the late-term clinical manifestations differ, these types of diseases may have a common foundation [15]. Some scholars have named them as placental ischemic diseases [16]. Pregnancy outcome varies according to the extent of spiral artery remodeling. Partial remodeling disorder is prone to be complicated by preterm birth and FGR without pregnancy-induced hypertension; complete remodeling disorder is often accompanied with PE [17]. Placenta dysfunctions in different pregnancy stages also indicate different outcomes, such as infertility, spontaneous abortion, RSA, late abortion, spontaneous preterm birth (no rupture of membranes and premature rupture of membranes), PE, and FGR [18]. Gunnarsdottir et al. [11] have confirmed that patients with a history of more than two abortions were exposed to an increasing risk of placenta-derived pregnancy-related complications, which is consistent with our study results. In terms of FGR, the pathogenesis between early-onset and late-onset FGR differs. The early-onset FGR is caused by reduced density of trophoblastic vessels and impaired invasion ability in the second trimester [19], which is similar to that of FRSA. In this study, patients with FGR were subgrouped by their gestational weeks at the first diagnosis. We discovered that patients who suffered from FRSA had a significantly higher risk of early-onset FGR. Previous studies have shown that patients with early-onset PE may have early placental implantation defects and spiral artery remodeling disorders, further leading to placental ischemia. Late-onset PE is often related to maternal basic conditions, such as cardiovascular diseases and metabolic diseases [20]. Therefore, early-onset PE and recurrent miscarriage are more closely related. Although the incidences of early-onset and late-onset PE in study group were both higher than those in the control group, the difference in early-onset PE was not statistically significant. In view of the low incidence of PE and the limited sample size, further enrollment of PE patients is needed to identify the relationship between FRSA and early-onset PE.

History of multiple abortions and abortion curettages are confirmed to be high risk factors of placenta previa and placenta accreta [21]. But the relationship between gestational hypertension and placenta previa remains controversial. A meta-analysis by Ying et al. [22] revealed that patients with gestational hypertension have a reduced risk of placenta previa. In this study, no significant difference of placenta previa occurrence was observed between the two groups. Maybe increased incidence of PE provides some protective factors for placenta previa. Usta et al. [23] suggest that a history of multiple pregnancy will affect the incidence of placenta accreta. In this study, the incidences of placental accreta and placental increta/percreta were higher in the study group than in the control group, which is consistent with those previous studies.

### 4.2. Maternal Pregnancy Outcomes

This study shows that RSA is an independent risk factor for cesarean section, which is consistent with previous studies [3]. In our study group, the age differences and history of abortion lead to no-indication cesarean sections. Placenta previa, placenta implantation/penetration, and FGR raised the incidence of cesarean sections with absolute indications. Postpartum hemorrhage rate was significantly higher in the study group than in the control group. Previous studies have shown that PE is an independent risk factor for uterine contraction fatigue and postpartum hemorrhage after vaginal delivery [24], and placental abnormalities (placenta previa, implantation, or early ablation) and hypertension are closely related to severe postpartum hemorrhage [25, 26].

### 4.3. Perinatal Outcomes

For FRSA, possibly due to the limitations of traditional cytogenetic techniques, no abnormal chromosomes in couples or previous fetus' chromosomal submicroscopic abnormalities were found. Increased incidence of villi genome microdeletions or microrepetition will lead to abortion or fetal chromosomal abnormalities in subsequent pregnancies [27]. Previous studies also revealed that subsequent pregnancies of FRSA patients were prone to fetal structural abnormalities [28, 29], especially for those with advanced maternal age and a history of more than three miscarriages. In this study, the proportion of neonatal birth defects in the study group increased accordingly.

Recent animal studies have shown that adverse factors in early and mid-term pregnancy affect pregnancy outcomes seriously, especially on the placenta and fetus. In late-term pregnancy, maternal involvement is mainly affected by adverse factors [30]. It can be inferred that unexplained abortions and abortions of failed effective interventions might be affected prenatally and continued till mid-pregnancy. Placenta and fetus suffered the most, leading to placental-derived pregnancy-related complications, such as late-onset PE, oligohydramnios, early-onset FGR, late abortion, placenta accreta, placenta increta, and placenta percreta. However, the numbers for the outcomes of early-onset FGR and placenta increta and placenta percreta are too small, and our further research will focus on those aspects.

The recent impact on fetuses is an increasing risk of perinatal death, FGR, small for gestational age fetus, and preterm birth [31, 32]. Current studies suggest that placenta dysfunctions have a profound impact on fetal long-term health, such as cardiovascular diseases, hypertension, obesity, nervous system dysfunctions, and psychological behavior abnormalities [33, 34]. In this study, we mainly focused on the near-term influences on fetal health. The incidence of small for gestational age fetus, perinatal death, and neonatal severe asphyxia in the study group was higher than that in the control group, but the difference was not statistically significant. Small sample size, excellent capacity of prenatal care, and after-birth pediatric treatment for high risk pregnancies in our hospital may contribute to this situation. Enlarged sample size will be needed and long-term fetus health conditions should also be considered in future researches.

### 4.4. Innovations of This Study

To the best of our knowledge, it is the first study that subdivided PE and FGR patients to identify the correlations between FRSA and early-onset PE and FGR. In addition, the second-trimester abortion rate in patients who experienced FRSA is 18.059 times of that in those without previous FRSA. The current studies on maternal-fetal interface immunity revealed that both second-trimester abortion and spontaneous preterm birth, similar to those of PE and FGR, suffered from abnormal immune cell function and cell number, leading to disorders of maternal-fetal tolerance and uterine spiral artery reconstructions [26]. Recent reports have shown that about 50~65% of women with unexplained spontaneous abortion have at least one inherited or acquired prethrombotic state (PTS) [35]. In PTS, FVL mutation raises the risk of early miscarriages and early intrauterine fetal deaths [36]. Meta-analysis has shown that late spontaneous abortion is closely related to factors V and II (prothrombin) mutations and to the lack of protein S induced congenital thrombosis [37]. Clinically, patients with RSA are not recommended for routine hereditary thrombosis test, in that mutations of factors V and II (prothrombin) in domestic Han population are rare. Therefore, tests of FVL, coagulation factor VIII, antithrombin, and protein C are only recommended for patients with a thrombosis family history and who suffered from unexplained FRSA. Late abortions should also be alerted.

### 4.5. Limitations of This Study

Sample selection bias in this retrospective study is inevitable. The diagnosis of unexplained abortion in the study group is exclusive. Due to the complexity of pathogenesis and the lack of specific clinical manifestations, FRSA diagnosis was mainly based on laboratory results. However, the test stability and standard are not unified among different laboratories, the exclusion of abortion causes was incomplete, and false positive diagnosis could not be avoided. Meanwhile, as our hospital is a university-affiliated high-class hospital and regional intense care referral center, patients in the control group could also be high risk pregnancies which to some extent weakened the difference between the two groups. In future study, with the standardization of RSA diagnosis and screening, a multicenter study should be performed for more objective and precise investigations on pregnancy outcomes.

For patients with a history of FRSA, this study shows an increased incidence of adverse maternal-placental-fetal outcomes for subsequent pregnancy, especially for placenta-derived pregnancy-related complications. Late abortions, oligohydramnios, and PE are all independent risk factors for FRSA. For such pregnant women, perinatal care should focus on the monitoring of maternal-placental-fetal and neonatal conditions from prepregnancy to postpartum period. All of the therapies should be implemented both generally and individually. Specific steps are as follows: further exploration of abortion causes during prepregnancy or early pregnancy, especially for those with unexplained abortions; development of individualized prenatal care mode and monitoring treatment program in combination with maternal high risk factors and abortion check results; focusing on placental-fetal-maternal aspects as well as their interactions for perinatal care; individualized treatments for early intervention of related complications; dynamic monitoring measures and effective and safe treatments; effective prevention of adverse pregnancy outcomes during delivery; and postpartum monitoring and treatment and follow-up. All of the above can fundamentally reduce the incidence of mother and child complications and improve the adverse pregnancy outcomes.

## Figures and Tables

**Table 1:** Comparison of clinical characteristics between the two groups.

Characteristics	Study group (*N* = 164)	Control group (*N* = 328)	*P* value
Maternal age (years)	33.97 ± 4.25	30.48 ± 3.65	<0.001
Gravidity	3.9 ± 1.1	2.2 ± 1.0	<0.001
Gestational age (w)	35.7 ± 5.2	38.2 ± 2.1	<0.05
Birth weight (g)	2676 ± 1014	3245 ± 70	<0.001
Body mass index	23.8 ± 3.3	24.2 ± 2.9	>0.05
Proportion of primiparous women	145 (88.4%)	283 (86.2%)	>0.05
Received ART	17 (10.3%)	26 (7.9%)	>0.05
Other adverse pregnancy histories	11 (6.7%)	19 (5.8%)	>0.05

**Table 2:** Comparison of maternal pregnancy outcomes between the two groups.

Characteristics	Study group	Control group	OR	95% CI	*P* value
	*N* (%)	*N* (%)			
Cesarean section	105 (64.02)	152 (46.34)	2.06	1.40–3.03	<0.001
Postpartum hemorrhage	17 (10.37)	18 (5.49)	1.99	0.10–3.98	<0.05

**Table 3:** Comparison of placenta diseases between the two groups.

Characteristics	Study group *N* (%)	Control group *N* (%)	OR	95% CI	*P* value
*Placenta-derived maternal complications*					
Early-onset PE	7 (4.27)	6 (1.83)	2.39	0.791–7.24	0.196
Late-onset PE	17 (10.37)	9 (2.74)	4.1	1.79–9.41	0.002
Oligohydramnios	17 (10.37)	7 (2.13)	5.3	2.15–13.06	<0.001
Early-onset FGR	8 (4.88)	2 (0.61)	8.36	1.75–39.83	0.005
Late-onset FGR	3 (1.83)	5 (1.52)	1.2	0.28–5.10	1
Placental abruption	3 (1.83)	1 (0.30)	6.09	0.639–59.04	0.241
Late abortion	16 (9.76)	3 (0.91)	11.71	3.36–40.81	<0.001
Preterm birth	8 (4.88)	8 (2.44)	2.051	0.76–5.57	0.15
Very preterm birth	14 (8.54)	23 (7.01)	1.24	0.62–2.47	0.546
*Placenta diseases*					
Placenta previa	21 (12.80)	30 (9.15)	1.46	0.81–2.64	0.209
Placenta accreta	22 (13.41)	9 (2.74)	5.49	2.47–12.23	<0.001
Placenta increta and placenta percreta	7 (4.27)	2 (0.61)	7.268	1.49–35.39	0.012

**Table 4:** Comparison of late-onset PE between the two groups.

Variable	Late-onset PE					
	Unadjusted			Adjusted		
	OR	95% CI	*P* value	OR	95% CI	*P* value
Maternal age	4.1	1.79–9.41	0.002	3.2	1.34–7.63	0.009

**Table 5:** Comparison of adverse fetal and neonatal outcomes between the two groups.

Characteristics	Study group *N* (%)	Control group *N* (%)	OR	95% CI	*P* value
Small for gestational age	7 (4.27)	5 (1.52)	2.88	0.90–9.22	0.121
Perinatal death	3 (1.83)	2 (0.61)	3.04	0.50–18.36	0.427
Neonatal severe asphyxia	1 (0.61)	1 (0.30)	2.01	0.13–32.28	0.627
Birth defects of newborns	12 (7.32)	3 (0.91)	8.55	2.38–30.75	<0.001

**Table 6:** Adverse pregnancy outcomes related to recurrent spontaneous abortions.

Adverse outcomes	Regression coefficient *β*	Standard error	Wald *χ*^2^	*P*	OR	95% CI
PE	1.306	0.348	14.102	0	3.69	1.87–7.30
Oligohydramnios	1.531	0.471	10.565	0.001	4.62	1.84–11.64
Late abortion	2.461	0.637	14.925	0	11.71	3.36–40.81
Cesarean section	0.531	0.242	4.825	0.028	1.7	1.06–2.73
